# Association of TET3 epigenetic inactivation with head and neck cancer

**DOI:** 10.18632/oncotarget.25333

**Published:** 2018-05-11

**Authors:** Kiyoshi Misawa, Atsushi Imai, Daiki Mochizuki, Masato Mima, Shiori Endo, Yuki Misawa, Takeharu Kanazawa, Hiroyuki Mineta

**Affiliations:** ^1^ Department of Otolaryngology, Head and Neck Surgery, Hamamatsu University School of Medicine, Shizuoka, Japan; ^2^ Department of Otolaryngology, Head and Neck Surgery, Jichi Medical University, Tochigi, Japan

**Keywords:** TET family genes, DNA methylation, HNSCC, DFS, site-specific analysis

## Abstract

The aim of this study was to clarify the epigenetic regulation of ten eleven translocation protein (TET) family genes, which can provide insights into the mechanisms of tumorigenesis and the risk of disease recurrence in head and neck squamous cell carcinoma (HNSCC). We generated methylation profiles of *TET1*, *TET2* and *TET3* genes in tumor samples obtained from 233 patients with HNSCC; these included 57 hypopharynx, 44 larynx, 69 oral cavity, and 63 oropharynx tumor samples. The mRNA expression and promoter DNA methylation of *TET* family genes were examined via quantitative RT-PCR and methylation-specific PCR, respectively. Promoter methylation was compared with various clinical characteristics and the *TET* methylation index (TE-MI). The TE-MI, representing the number of methylation events in *TET* family genes, was positively correlated with alcohol consumption (P = 0.004), high-risk human papilloma virus (HPV) status (P = 0.004) and disease recurrence (P = 0.002). The simultaneous methylation analysis of *TET* family genes was correlated with reduced disease-free survival in unfavorable event groups (log-rank test, P = 0.026). In the multivariate Cox proportional hazards analysis, *TET3* methylation in T1 and T2 tumor stages, oropharyngeal cancer, and oral cancer patients exhibited high association with poor survival (hazard ratio: 2.64, P = 0.014; 3.55, P = 0.048; 2.63, P = 0.028, respectively). A joint analysis of the tumor suppressor gene methylation index showed a significant trend toward a higher TE-MI. The methylation status of *TET3* was independently associated with aggressive tumor behavior and a global effect on DNA methylation status in HNSCC.

## INTRODUCTION

Head and neck squamous cell carcinomas (HNSCC) constitute an anatomically heterogeneous group of solid tumors arising from the nasopharynx, oral cavity, oropharynx, hypopharynx, and larynx [1]. Major risk factors for HNSCC include sex, tobacco smoking, alcohol consumption, and oncoviral infection [2]. At least 50% of patients with locally advanced HNSCC develop local lymph node failure or distant failure in the lung, which is usually detected within the first 2 years of treatment [3]. The present standard management strategies include constructive and multimodal treatments such as surgery, radiotherapy, and chemotherapy. Despite these aggressive treatments, the long-term survival rates are poor and remain between 40% and 50% [4]. Therefore, molecular classification of HNSCCs is required to provide prognostic as well as mechanistic information to improve patient care.

Aberrant promoter methylation, an important hallmark of cancer cells, is considered a major mechanism underlying the inactivation of tumor-related genes. Several studies have reported that the promoter methylation of tumor suppressor genes represents a common mechanism of transcriptional silencing in HNSCC [5]. DNA methyltransferases (DNMTs) play an important role in genomic integrity, the disruption of which may result in chromosomal instability and tumor progression [6]. DNMT levels, especially those of DNMT3A and DNMT3B, are often increased in various cancer tissues and cell lines, which may partly account for the hypermethylation of CpG-rich regions in tumor suppressor gene promoters [7]. In HNSCC, the increased methylation observed in HPV-positive tumors may be partially explained by the higher expression of *DNMT3A*, compared to that in HPV-negative cells [8].

The ten eleven translocation protein (TET) might function as a 5-methylcytosine (5mC) oxidase and potentially as a DNA demethylase [9]. TET belongs to a family of three proteins, namely TET1, TET2, and TET3, which catalyze the successive oxidation of 5mC to 5-hydroxymethylcytosine (5hmC), 5-formylcytosine (5fC), and 5-carboxylcytosine (5caC) [10–12]. Mutations of *TET* genes were found in 0.1–10% of major types of cancer [13]. *TET* gene inactivation may have broad implications for the formation of many solid tumors [14]. *TET1* methylation appears to be an early event during colorectal cancer tumorigenesis and is associated with a global effect on the DNA CpG methylation status [15]. However, a systematic study of the epigenetic and transcriptional regulation of *TET* family genes in most human cancers is still needed. Simultaneous analyses of the methylation status of *TET* family genes are important for predicting tumorigenesis, biological behavior, and the development of future targeted therapies.

The aim of this study was to resolve the frequent promoter methylation of *TET* family genes in a large set of primary tumors. This appears to be the dominant mechanism for the inactivation of *TET* family genes in cancers. Furthermore, we determined the methylation status of *TET* family genes in HNSCC to evaluate their clinical significance as prognostic biomarkers for recurrence risk and survival. All three *TET* family genes were examined, as was the relationship between the methylation of *TET* family genes and various clinical characteristics. We attempted to determine whether HNSCC primary tumors originating from different anatomic sites (hypopharynx, larynx, oral cavity, and oropharynx) exhibited similar DNA methylation changes, or whether DNA methylation events were specific to the anatomic site.

## RESULTS

### Initial screening: expression and promoter methylation in *TET* family genes in HNSCC cell lines

**Figure 1:**
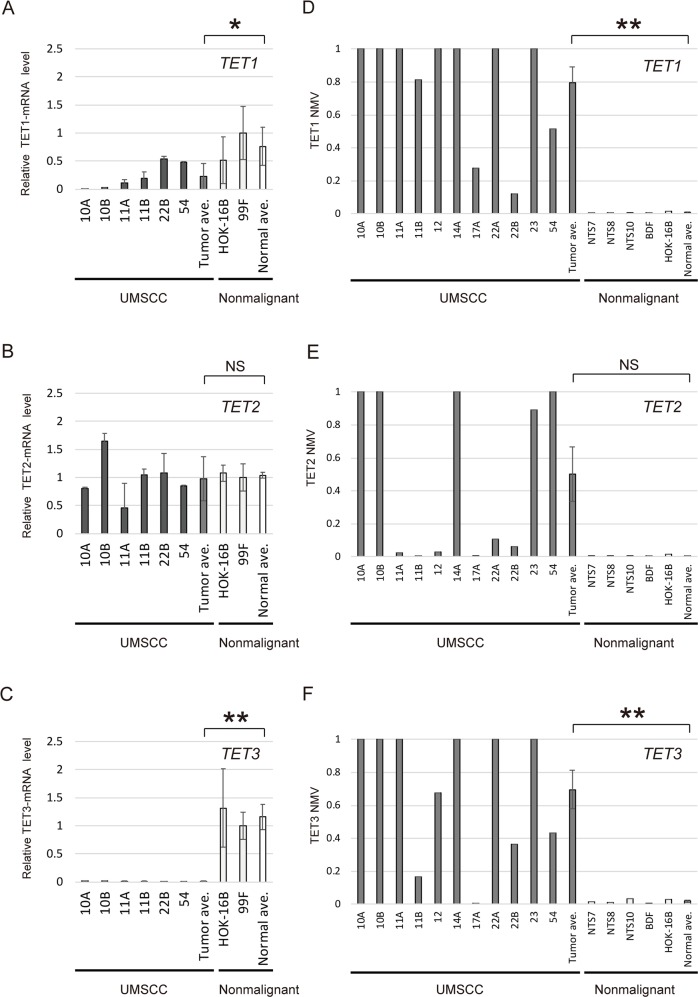
mRNA expression and promoter methylation of *TET* family genes in HNSCC cell lines The relative mRNA levels for **(A)** *TET1* and **(C)** *TET3* were lower in cancer cell lines than in normal cell lines (P = 0.041 and P < 0.001, respectively). **(B)** Expression of *TET2*, showing no significant association with cancer or normal cell lines (P = 0.842). Mean NMVs for the **(D)** *TET1*, **(E)** *TET2* and **(F)** *TET3* promoters, showing higher levels in cancer cell lines than in normal tonsil samples (P < 0.001, P = 0.059 and P = 0.002, respectively). NTS: normal tonsil sample. ^*^P < 0.05. ^**^P < 0.01. The data are shown as the mean ± SE.

Quantitative reverse transcription polymerase chain reaction (Q-RT-PCR) analysis of *TET1* and *TET3* transcripts from UM-SCC cell lines (UM-SCC-10A, -10B, -11A, -11B, -22B, and -54) revealed significantly lower expression of these genes in cancer cell lines than in normal cell lines (P = 0.041 and P < 0.001, respectively) (Figure 1A and 1C). Moreover, in the quantitative methylation-specific PCR (Q-MSP) analysis, the normalized methylation value (NMV) for the *TET1* and *TET3* gene promoters tended to be higher in cancer cells than in normal tonsil samples and normal cell lines (P < 0.001 and P = 0.002, respectively) (Figure 1D and 1F). There was no significant difference in *TET2* expression and methylation between cancer cell lines and normal cell lines (Figure 1B and 1E). *TET1*, *TET2* and *TET3* promoter hypermethylation showed highly discriminative ROC curve profiles, which clearly distinguished HNSCC from normal mucosal tissues (AUROC = 0.6694, AUROC = 0.5968, and AUROC = 0.6559, respectively). A DNA sample was classified as positive when the NMV exceeded 0.0471, 0.1004 and 0.1337 for *TET1, TET2* and *TET3*, respectively. The cutoff NMV was chosen from the ROC curve to maximize sensitivity and specificity (Supplementary Figure 1). In total, 233 primary HNSCC samples and 128 adjacent normal mucosal tissues were obtained from surgical specimens for methylation screening. (Supplementary Table 1).

### Analysis of the methylation status of *TET* family genes in primary samples

**Figure 2:**
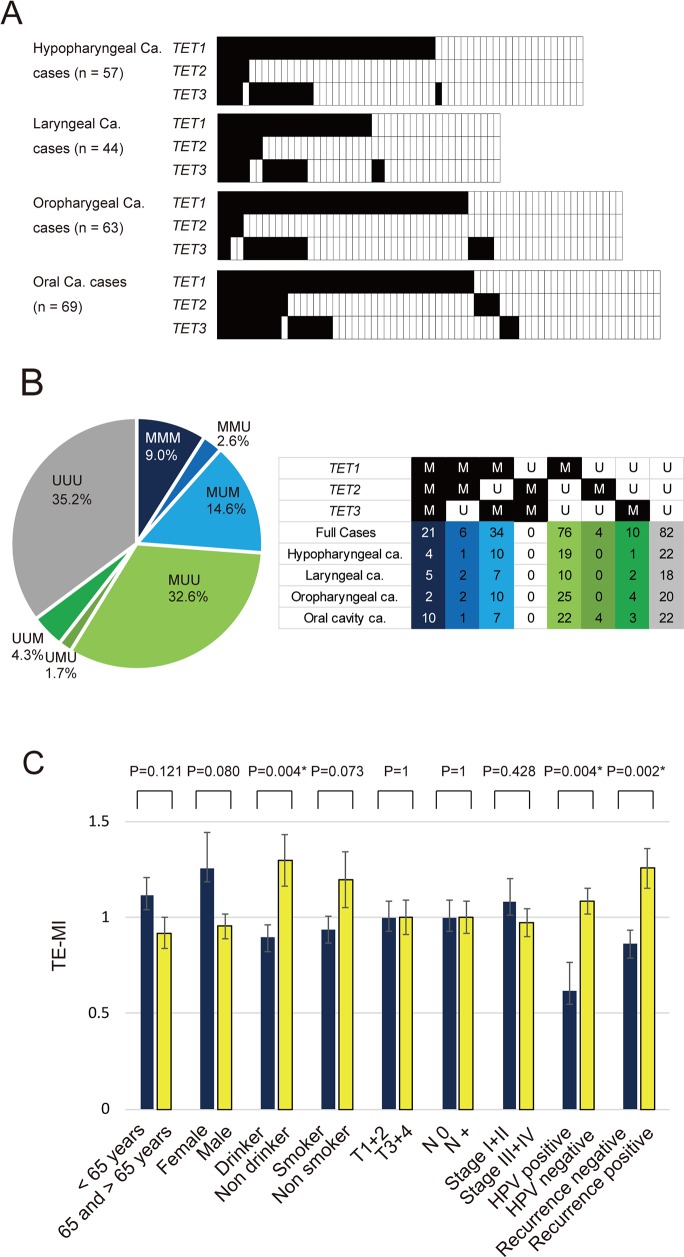
Summary of the promoter methylation status of *TET* family genes in 233 HNSCC samples **(A)** Methylation rate comparisons of the promoters of three genes (*TET1*, *TET2* and *TET3*) in patients with hypopharyngeal cancer, laryngeal cancer, oropharyngeal cancer, and oral cancer. Filled boxes indicate the presence of methylation, and open boxes indicate the absence of methylation. **(B)** Distribution of promoter methylation in *TET* family genes. Methylation was observed in the promoters of all three *TET* genes (MMM), two of three *TET* genes (UMM, MMU, MUM), only one *TET* gene (UUM, UMU, MUU) and none of the *TET* genes (UUU) in 9.0%, 17.2%, 38.6%, and 35.2% of the tumors, respectively. **(C)** The mean TE-MI for the different groups were compared using Student's *t* test. ^*^P < 0.05. M: methylated; U: unmethylated. The data are shown as the mean ± SE.

Q-MSP was used to assess the aberrant promoter methylation status of *TET* family genes in tumors from the hypopharynx (n = 57), larynx (n = 44), oropharynx (n = 63), or oral cavity (n = 69). *TET1* was methylated in 34 (59.6%), *TET2* in 5 (8.8%), and *TET3* in 15 (26.3%) of the 57 hypopharyngeal cancers examined. In laryngeal cancers, the frequency of hypermethylation was 54.5% for *TET1*, 15.9% for *TET2*, and 31.8% for *TET3*. Moreover, the frequency of promoter methylation in oropharyngeal cancers was 61.9% for *TET1*, 6.3% for *TET2*, and 25.4% for *TET3*. Among 69 cases of oral cancer, the frequency of hypermethylation was 58.0% for *TET1*, 21.7% for *TET2*, and 29.0% for *TET3* (Figure 2A). The distribution across all tumor types of promoter methylation in *TET* family genes is shown in Figure 2B. Methylation was observed in the promoters of all three *TET* genes (MMM), two of the three *TET* genes (UMM, MMU, MUM), only one *TET* gene (UUM, UMU, MUU) and none of the *TET* genes (UUU) in 9.0%, 17.2%, 38.6%, and 35.2% of the tumors, respectively (Figure 2B).

### Association between methylation in *TET* family genes and clinicopathological characteristics

The characteristics and clinicopathological features of patients, including the age at diagnosis, sex, smoking habit, alcohol consumption, tumor staging, lymph node status, clinical stage, and HPV status are summarized in Table 1. TE-MI was defined as the number of methylated genes in each sample. The mean differences in TE-MI according to the age of onset, sex, smoking habit, alcohol consumption, tumor size, lymph node status, clinical stage, HPV status, and recurrence are illustrated in Figure 2C. TE-MI was significantly lower in drinkers (0.90 ± 0.88) than in non-drinkers (1.3 ± 1.05, P = 0.004), as well as in HPV-positive cases (0.62 ± 0.94) when compared to HPV-negative cases (1.08 ± 0.93, P = 0.004). Specifically, TE-MI was significantly higher in recurrent (1.26 ± 0.95) compared with nonrecurrent (0.86 ± 0.91) tumor cases (P = 0.002). No significant differences in methylation index (MI) were observed regarding the age of onset, sex, smoking status, tumor stage, lymph node status, or clinical stage (Figure 2C). For hypopharyngeal, laryngeal and oral cavity cancers, there was no significant association with clinicopathological characteristics (Supplementary Figure 2A, 2B, 2D). Among oropharyngeal cancers, TE-MI was significantly lower in female (0.40 ± 0.70) than in male (1.04 ± 0.78; P = 0.020) patients, as well as in non-drinkers (0.54 ± 0.66; P = 0.043) relative to drinkers (1.04 ± 0.81) (Supplementary Figure 2C).

**Table 1:** *TET1*, *TET2* and *TET3* gene methylation status in HNSCC primary samples

Patient and tumor characteristics	Methylation status								
	*TET1*			*TET2*			*TET3*		
	Present (137)	Absent (96)	*P*-value^a^	Present (31)	Absent (202)	*P*-value^a^	Present (65)	Absent (168)	*P*-value^a^
*Age*									
Under 65 (97)	58	39	0.893	12	85	0.845	29	68	1
65 and older (136)	79	57		19	117		36	100	
*Gender*									
Female (35)	19	16	1	6	29	1	13	22	1
Male (198)	118	80		25	173		52	146	
*Smoking status*									
Smoker (177)	102	75	0.538	24	153	1	55	122	0.061
Non smoker (56)	35	21		7	49		10	46	
*Alcohol exposure*									
Ever (173)	103	70	1	23	150	1	53	120	0.134
Never (60)	34	26		8	52		12	48	
*Tumor size*									
T1-2 (116)	72	44	0.352	18	98	0.342	28	88	0.243
T3-4 (117)	65	52		13	104		37	80	
*Lympho-node status*									
N0 (99)	52	47	1	13	86	1	29	70	1
N+ (134)	85	49		18	116		36	98	
*Stage*									
I, II (60)	32	28	1	8	52	1	17	43	1
III, IV (173)	105	68		23	150		48	125	
*HPV status*									
Positive (42)	28	14	0.300	5	37	1	15	27	1
Negative (191)	109	82		26	165		50	141	

^a^Fisher's exact probability test. ^*^P < 0.05.

### Methylation levels of 13 tumor suppressor genes and *TET* family genes in cancer tissues

**Figure 3:**
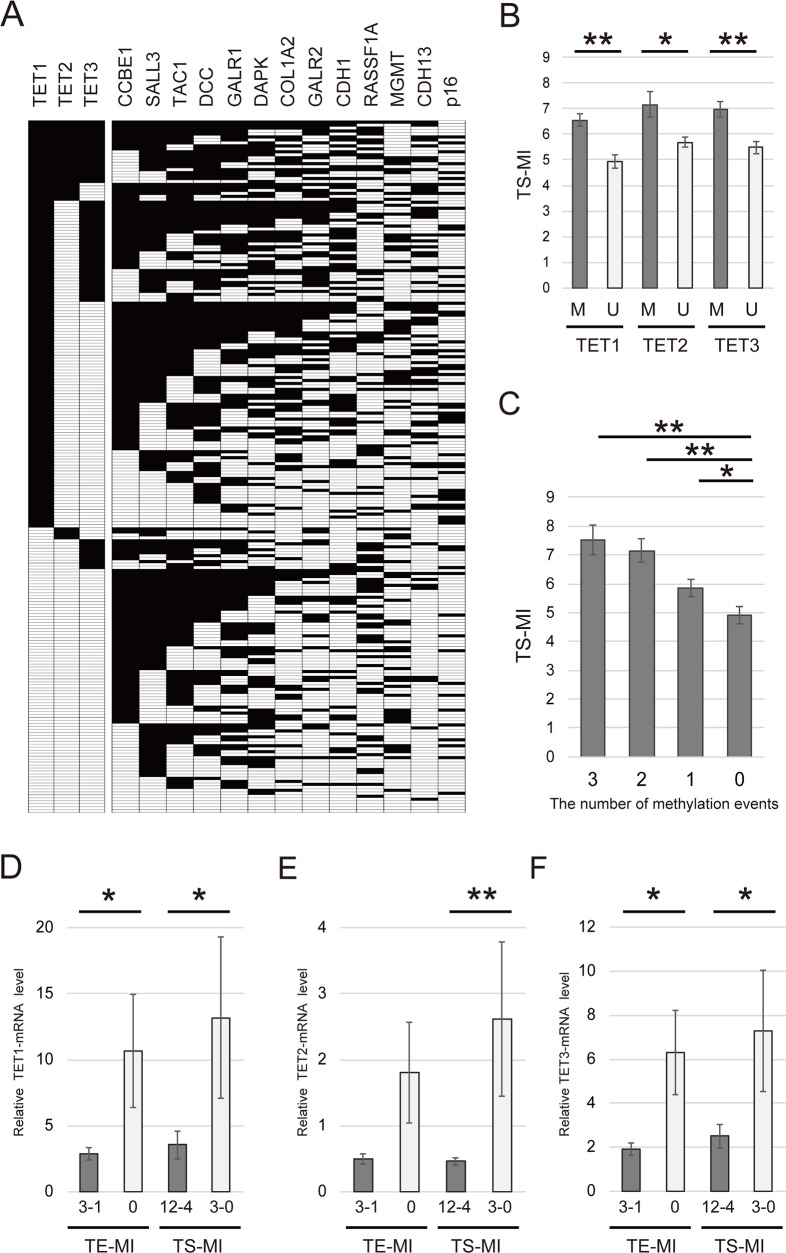
Comparison of methylation rates for 13 tumor suppressor genes and *TET* family genes in 233 primary HNSCC samples **(A)** Distribution of promoter methylation in *TET* family genes and 13 tumor suppressor genes. Filled boxes indicate the presence of methylation, and open boxes indicate the absence of methylation. **(B)** Correlation between the methylation status of TS-MI and TETs in patients with HNSCC. M: methylated; U: unmethylated. **(C)** Combined analyses involving the methylation status of TS-MI and *TET* family genes. The number of methylation events was indicated for hypermethylated *TET* family genes. The mean TS-MI for the different groups was compared using Student's *t* test. **(D)** *TET1* mRNA levels were significantly higher in groups with lower TE-MI and TS-MI (P = 0.045 and P = 0.024, respectively). **(E)** *TET2* mRNA levels were higher in groups with lower TE-MI compared to those with higher TE-MI (P = 0.055). *TET2* mRNA levels were significantly higher in groups with lower TS-MI than in those with higher TS-MI (P = 0.004) **(F)** *TET3* mRNA levels were significantly higher in groups with lower TE-MI and TS-MI (P = 0.012 and P = 0.014, respectively). ^*^P < 0.05. ^**^P < 0.001. The data are shown as the mean ± SE.

The 13 tumor suppressor genes (TS-MI) was defined as the number of methylated genes in each sample (Figure 3A). The mean differences in TS-MI based on the methylation status of *TET* family genes are illustrated in Figure 3B. Specifically, the TS-MI was significantly higher in patients with *TET1* methylation (6.55 ± 2.79) than in those with *TET1* unmethylation (4.94 ± 2.56, P < 0.001), in patients with *TET2* methylation (7.16 ± 2.81) than in those with *TET2* unmethylation (5.69 ± 2.76, P = 0.006) and in those with *TET3* methylation (6.95 ± 2.45) than in those with *TET3* unmethylation (5.48 ± 2.83, P < 0.001) (Figure 3B). Joint analysis of the methylation status of *TET1*, *TET2*, and *TET3* showed a significant trend toward higher TS-MI as the number of *TET* methylation events increased. This analysis revealed that TS-MI was significantly higher in patients with 3 (7.52 ± 2.29), 2 (7.15 ± 2.67), and 1 (5.84 ± 2.80) events than in patients with 0 events (4.90 ± 2.60, P < 0.001, P < 0.001, and P = 0.024, respectively) (Figure 3C).

### Expression and methylation index of *TET* family genes in HNSCC specimens

We next examined the mRNA levels of *TET* family genes in HNSCC specimens by Q-RT-PCR. *TET1* expression was significantly higher in TE-MI and TS-MI groups exhibiting lower methylation levels (P = 0.044 and 0.024, respectively) (Figure 3D). The expression of *TET2* was not associated with TE-MI (P = 0.055). However, *TET2* expression was correlated with TS-MI (P = 0.004) (Figure 3E). *TET3* expression was significantly correlated with both TE-MI and TS-MI (P = 0.012 and 0.014, respectively) (Figure 3F).

### Kaplan-Meier estimates

**Figure 4:**
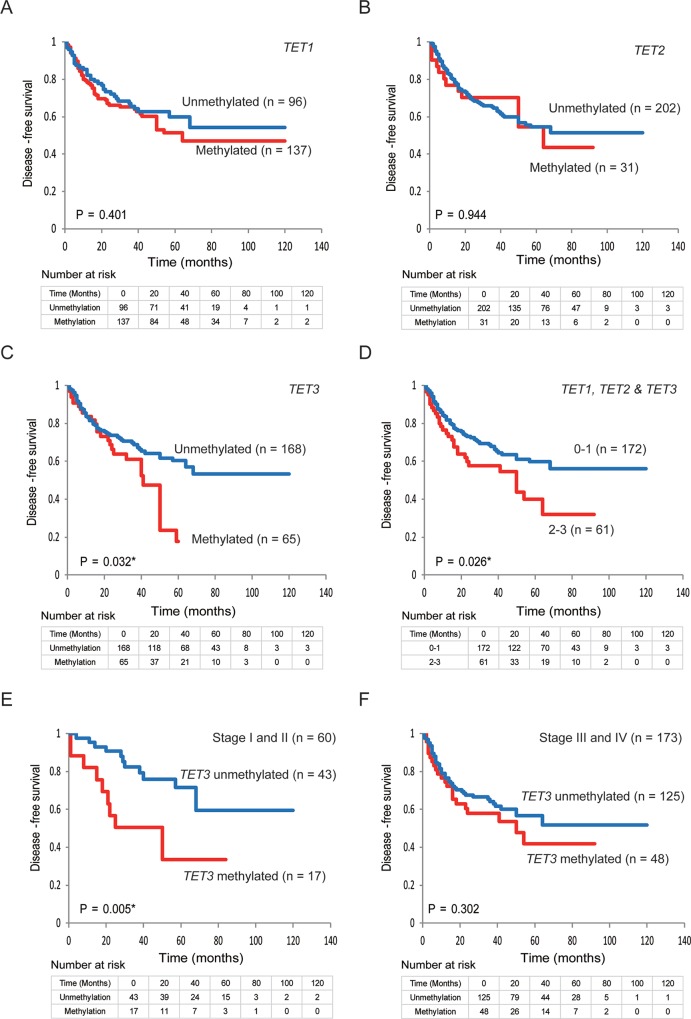
Kaplan-Meier survival curves for patients with HNSCC based on *TET* family gene methylation status Kaplan-Meier survival curves based on *TET* promoter methylation status in patients with HNSCC. Disease-free survival according to **(A)** *TET1* methylation status; **(B)** *TET2* methylation status; **(C)** *TET3* methylation status; **(D)** *TET1, TET2,* and *TET3* methylation status; **(E)** *TET3* methylation status in stage I and II patients; **(F)** and *TET3* methylation status in patients with stage III and IV cancers.

Kaplan-Meier plots indicated that the methylation status of *TET* family genes was correlated with disease-free survival (DFS) (Figure 4A–4F). The Kaplan–Meier survival curves for the 233 patients with HNSCC according to the methylation status of the *TET* family gene promoters are shown in Figure 4A–4C. No correlation with DSF time was observed in patients with methylated (compared with unmethylated) *TET1* and *TET2* promoters (log-rank test; P = 0.401 and P = 0.944, respectively) (Figure 4A and 4B). A shorter DFS time was observed in patients with methylated *TET3* promoters, compared with those with unmethylated *TET3* promoters (log-rank test, P = 0.032) (Figure 4C). The DFS rate in the cases with 2–3 methylated genes was 32.1%, as compared with 56.2% in the 0–1 methylation group (log-rank test, P = 0.026) (Figure 4D). Among the 60 patients with stage I and II HNSCC, those with a methylated *TET3* promoter had a shorter DFS time than those with an unmethylated *TET3* promoter (log-rank test, P = 0.005) (Figure 4E). However, among 173 patients with stage III and IV HNSCC, the DFS rate in patients with *TET3* methylation was 41.8%, as compared with 51.9% in the *TET3* unmethylated group (log-rank test, P = 0.302) (Figure 4F). No increase in the risk of recurrence was observed according to the hypermethylation status of any of the *TET* genes studied or for any other combination of hypermethylated genes (Supplementary Figure 3).

### Prognostic value of promoter hypermethylation in *TET* family genes

The association between methylation and the risk of recurrence was estimated via a multivariate analysis by using a Cox proportional hazards model adjusted for age, HPV status, smoking status, alcohol consumption, and clinical stage. In patients with a methylated *TET3* promoter (65/233, 27.9%), the adjusted odds ratio (OR) for recurrence was 1.63 (95% confidence interval [CI]: 1.02–2.61, P = 0.040). In patients with T1 and T2 tumor stage, *TET3* methylation showed a significant association with the OR for recurrence (OR = 2.64, 95% CI: 1.21–5.75, P = 0.014).

**Figure 5:**
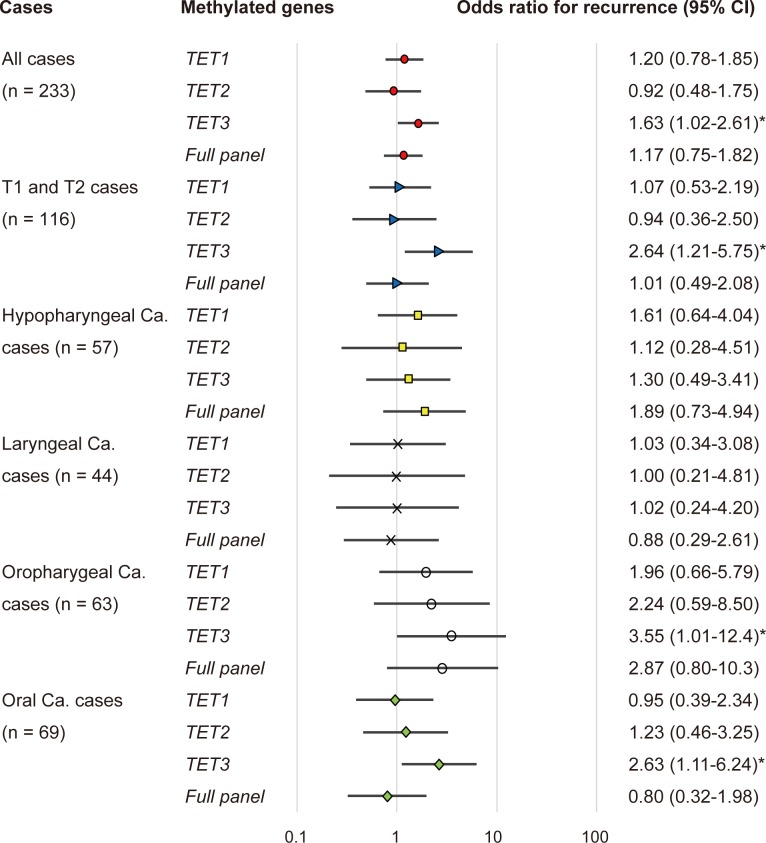
Odds ratios for recurrence based on the Cox proportional hazards model adjusted for age (65 years & older vs. < 65 years), HPV status, smoking status, alcohol exposure, and tumor stage (I, II or III, IV) Cox proportional hazards model, revealing the estimated odds of recurrence associated with *TET1, TET2* and *TET3* methylation; CI: confidence interval. ^*^: P < 0.05.

The ORs for recurrence were also determined based on the tumor origin for four sites in this study: the hypopharynx, larynx, oropharynx, and the oral cavity. For patients with oropharyngeal cancers with a methylated *TET3* promoter, the OR was 3.55 (95% CI: 1.01–12.44; P = 0.047). In patients with oral cancer and a methylated *TET3* promoter, the adjusted OR for recurrence was 2.63 (95% CI: 1.11–6.24, P = 0.027) (Figure 5).

### External validation of results using the cancer genome atlas (TCGA) database

Aberrant promoter methylation in *TET* family genes was detected in 516 HNSCC samples compared with 50 normal samples (Supplementary Figure 4). The average β values for *TET3* methylation were significantly higher in HNSCC samples than those in normal samples (P < 0.001), whereas those for *TET1* and *TET2* were not.

## DISCUSSION

This is the first study addressing the promoter CpG methylation-mediated silencing of *TET* family genes in HNSCC, which in turn leads to an increase in global methylation in tumor tissues. Clarifying the epigenetic regulation of *TET* family genes can provide insights into the mechanisms of tumorigenesis and the risk of disease recurrence in HNSCC. Additionally, our site-specific analysis found that hypermethylation of CpG islands in the *TET3* promoter was independently associated with aggressive tumor behavior in oropharyngeal and oral cancers.

We found that *TET1* and *TET3* expression was lower in HNSCC than in normal cells and was associated with the level of promoter methylation. When normal cells are exposed to environmental carcinogens (e.g., chemical carcinogens and oncogenic viruses), DNMTs catalyze higher levels of DNA CpG methylation (5mC) [16, 17]. Elevated levels of 5mC at tumor suppressor gene (TSG) promoters lead to TSG silencing and functional inactivation, ultimately contributing to tumor initiation or progression [18]. Growing evidence suggests that impairment of TET-mediated DNA demethylation may contribute to oncogenesis [19]. Loss of *TET* activation through promoter methylation occurs in tumor cells, successively increasing 5mC levels and promoting TSG inactivation [18, 19]. The current study has shown that a similar phenomenon occurs in HNSCC tissues. Thus, the epigenetic inactivation of *TET* family genes is likely to be common in cancers, and to play an important role in carcinogenesis. Simultaneous analysis of the *TET* methylation status will allow us to better predict tumor-related events, assess biological behavior, and design targeted therapies for HNSCCs.

Exposure to several carcinogens, such as HPV, Helicobacter pylori, tobacco, and alcohol, has been associated with epigenetic gene inactivation in human cancers, e.g., those of the head and neck, esophagus, stomach, and cervix [20, 21]. Recently, oncogenic viruses such as HPV and EBV have been shown to evoke cancerous changes in the DNA methylome of the cell by increasing the activity of DNMTs, which methylate the DNA of the host genome as part of the tumorigenic pathway [22, 23]. Additionally, this increase in DNMT activity may lead to global DNA hypermethylation upon the loss of *TET* activity [13].

The TET family contains three members, i.e., TET1, TET2, and TET3, all of which share a high degree of homology with their C-terminal catalytic domain, suggesting that this family of enzymes may participate in a potentially novel mechanism underlying the regulation of DNA methylation [11, 24, 25]. The TET family consists of key molecules closely connecting 5mC and 5hmC [26]. Wild-type *TET2* showed strong 5mC oxidation activity, converting a large amount of 5mC into 5hmC and a significant amount of 5fC and 5caC. In contrast, mutation of *TET2* significantly decreased the enzymatic activity with a very low amount of 5hmC generated [27]. Thus, each TET may play a specific role depending on the cell type and the different sites of tumor development. A study of this type involving human specimens and utilizing high-throughput profiling platforms may be susceptible to measurement biases from a variety of sources. Our study is the first to suggest that the increased DNA methylation of *TET* family genes correlates with tumor progression and may promote the accumulation of aberrant methylation in head and neck cancers. Our analyses revealed that high *TET3* methylation in tumors predicts poorer survival. Our findings suggest that such methylation markers could be used in clinical practice to distinguish patients that may benefit from adjuvant therapy after the initial surgical treatment; however, additional prospective studies are required to validate these genes in other groups of patients with HNSCC.

Decreased *TET* family gene expression in malignant solid tumors is reportedly due to mutation or aberrant DNA methylation [15, 18, 28]. A study of hepatocellular carcinoma observed decreased expression of *TET1*, but not *TET2* and *TET3* [29]. Moreover, decreased *TET1* expression correlates with tumor progression and may serve as a potential prognostic biomarker in endometrial cancer [30]. Similarly, loss of 5hmC in gastric cancer was mainly correlated with the downregulation of *TET1* [31]. In addition, *TET2* promoter methylation, but not *TET2* mutations, may represent an alternative mechanism of pathogenesis in low-grade gliomas lacking IDH1/2 mutations [32]. *TET2* expression was significantly lower in esophageal squamous cell carcinoma and associated with 5hmC levels [33]. Furthermore, CpG methylation-induced silencing of *TET2* and *TET3* induced EMT-like progression and metastasis in melanoma [34]. Moreover, *TET3* functions as a potent tumor suppressor downstream of the TLX nuclear receptor to regulate growth and self-renewal in glioblastoma [35]. Missense and truncating mutations in *TET* genes are present in nearly all solid tumor types at a relatively low frequency [13]. In the TCGA cohort of HNSCC, *TET1*, *TET2* and *TET3* mutations were identified in 9 of 510 patients (1.8%), 8 patients (1.6%) and 8 patients (1.6%), respectively [36]. However, the role of *TET* family genes, especially *TET3*, in the tumorigenesis of HNSCC remains largely unknown. Our findings provide evidence that *TET3* methylation may represent a good biomarker for prediction of recurrence in early-stage head and neck cancers. Because increased frequency of DNA methylation in certain genes can determine the behavior of these tumors, it may be possible that HNSCC with *TET3* methylation exhibits unique clinicopathological features compared to that without *TET3* methylation. This finding may facilitate HNSCC screening and the development of surveillance algorithms.

In conclusion, *TET* family genes were identified as aberrantly methylated in patients with HNSCC. We demonstrated for the first time that *TET* mRNA is downregulated in HNSCC owing to DNA methylation; this may be a critical event in HNSCC progression. Importantly, the methylation patterns of these three genes in primary tumors may be used to identify patients with oral and oropharyngeal cancers that are at a higher risk of recurrence. The differences in global methylation patterns observed between *TET* methylation-positive and *TET* methylation-negative tumors, and their effects on the onset and progression of HNSCC, provide several testable hypotheses for further research.

## MATERIALS AND METHODS

### Tumor samples and cell lines

In total, 233 primary HNSCC samples were obtained from patients during surgery at the Department of Otolaryngology, Hamamatsu University School of Medicine. All patients provided written informed consent and the study protocol was approved by the Institutional Review Board of the Hamamatsu University School of Medicine. Clinical information, including age, sex, tumor site, smoking habit, alcohol consumption, tumor size, lymph node status, and stage grouping were obtained from the patients’ clinical records. The male:female patient ratio was 198:35. The mean age was 65.5 years (range = 32–92). Primary tumors were in the hypopharynx (n = 57), larynx (n = 44), oral cavity (n = 69), or oropharynx (n = 63). DNA and complementary DNA (cDNA) from 11 University of Michigan squamous cell carcinoma (UM-SCC) cell lines, 99F fibroblast and BDF fibroblast cell lines, and HOK-16B cells were provided by Dr. Thomas E. Carey of the University of Michigan.

### RNA extraction and Q-RT-PCR

Total RNA was isolated using an RNeasy Plus Mini Kit (Qiagen, Hilden, Germany), and cDNA was synthesized using a ReverTra Ace qPCR RT Kit (Toyobo, Tokyo, Japan). The mRNA levels of *TET1, TET2*, *TET3* and glyceraldehyde 3-phosphate dehydrogenase (*GAPDH*) were measured via Q-RT-PCR using SYBR Premix Ex Taq (Takara, Tokyo, Japan), the Takara Thermal Cycler Dice Real Time System TP8000 (Takara) and the primer sets presented in Supplementary Table 2. The data were analyzed using the ^ΔΔ^Ct method.

### Bisulfite treatment and Q-MSP analysis

Extraction and bisulfite conversion of genomic DNA from 233 primary HNSCC and 36 noncancerous mucosal samples were performed using the MethylEasy Xceed Rapid DNA Bisulfite Modification Kit (Takara) per manufacturer instructions [37]. The CpG island methylation levels in the promoters of the *TET1, TET2,* and *TET3* genes were determined via Q-MSP with the Takara Thermal Cycler Dice Real Time System TP800 (Takara); the primer sets are listed in Supplementary Table 2. A standard curve was constructed by plotting known concentrations of serially diluted EpiScope Methylated HeLa gDNA (Takara). The NMV was determined as follows: NMV = (target gene-S/target gene-FM)/(ACTB-S/ACTB-FM), where target gene-S and target gene-FM represent target gene methylation levels in the tumor sample and universal methylated DNA control, respectively, and ACTB-S and ACTB-FM represent *ACTB* (which encodes β-actin) methylation levels in the sample and control, respectively. The analysis was performed using Thermal Cycler Dice Real Time System TP800 software (version 1.03A), according to the manufacturer's instructions [38]. To analyze the methylation status of *CCBE1* [39], *SALL3* [40], *TAC1* [41], *DCC* [42], *GALR1* [43], *DAPK* [44], *COL1A2* [45], *GALR2* [43], *CDH1* [46], *RASSF1A* [46], *MGMT* [44], *CDH13* [47], and *p16* [46], primers and conditions were as previously described.

### Analysis of HPV status

The HPV status was evaluated using the HPV Typing Set (Takara Bio., Tokyo, Japan), a PCR primer set specifically designed to identify HPV genotypes -16, -18, -31, -33, -35, -52 and -58 in genomic DNA. The PCR HPV Typing Set method was performed according to the manufacturer's protocol. The PCR products were separated using 9% polyacrylamide gel electrophoresis and stained with ethidium bromide.

### Collection of publicly available data from TCGA

Data on aberrant DNA methylation from the TCGA (available in November 2017) were collected from MethHC, a DNA methylation and gene expression database for human cancers (http://methhc.mbc.nctu.edu.tw/php/index.php) [48]. The DNA methylation data were collected using the Infinium HumanMethylation450 platform (Illumina, Inc., San Diego, CA, USA) and are presented as β values.

### Data analysis and statistics

Q-MSP results and patient characteristics (age of onset, sex, alcohol consumption, smoking status, tumor size, tumor stage, clinical stage, lymph node status, and recurrence) were compared using Student's *t*-test. Receiver-operator characteristic (ROC) curve analyses were performed using the NMVs for 36 HNSCC and 36 adjacent normal mucosal samples and the Stata/SE 13.0 system (Stata Corporation, TX, USA). The area under the ROC curve indicated the optimal sensitivity and specificity cutoff levels for distinguishing between the methylation levels in normal and HNSCC tissues; the NMV thresholds were calculated for each target gene (Supplementary Figure 1). The cutoff values were used to determine the methylation frequencies of the target genes. The overall methylation values in individual samples were determined by calculating the MI. TE-MI and the methylation index of TS-MI were defined as the ratio of the number of methylated genes to the number of tested genes in each sample [38].

DFS was measured from the date of the initial treatment to the date of diagnosis with locoregional recurrence or distant metastasis. The Kaplan-Meier test was used to calculate survival probabilities, and the log-rank test was used to compare survival rates. The prognostic value of the methylation status was assessed by performing a multivariate Cox proportional hazards analysis adjusted for age (≥ 65 versus < 65 years), HPV status, smoking status, alcohol intake, and tumor stage (I and II versus III and IV). The Schoenfeld residuals test used in assessing the proportional hazard assumption was used to determine the goodness of fit [49]. Differences with P < 0.05 were considered significant. Statistical analyses were performed using StatMate IV software (ATMS Co. Ltd., Tokyo, Japan) and the Stata/SE 13.0 system (Stata Corporation, TX, USA).

## SUPPLEMENTARY MATERIALS FIGURES AND TABLES



## Abbreviations

TET: ten-eleven translocation
HNSCC: head and neck squamous cell cancer
TE-MI: *TET* methylation index
DNMTs: DNA methyltransferases
5mC: 5-methylcytosine
5hmC: 5-hydroxymethylcytosine
5fC: 5-formylcytosine
5caC: 5-carboxylcytosine
UM-SCC: University of Michigan squamous cell carcinoma
cDNA: complementary DNA
GAPDH: glyceraldehyde 3-phosphate dehydrogenase
NMV: normalized methylation value
ACTB: β-actin
HPV: human papilloma virus
TCGA: The Cancer Genome Atlas
ROC: Receiver operating characteristic
MI: methylation index
TS-MI: the methylation index of 13 tumor suppressor genes
DFS: disease-free survival
OR: odds ratio
CI: confidence interval
