# Supplementary material for: Association of TET3 epigenetic inactivation with head and neck cancer
Source: Oncotarget. 2018 May 11;9(36):24480–93. doi: 10.18632/oncotarget.25333 (PMC5966249; doi:10.18632/oncotarget.25333)
Supplement: Supplementary file 1 [file oncotarget-09-24480-s001.pdf]

Association of TET3 epigenetic inactivation with head and neck cancer

SUPPLEMENTARY MATERIALS

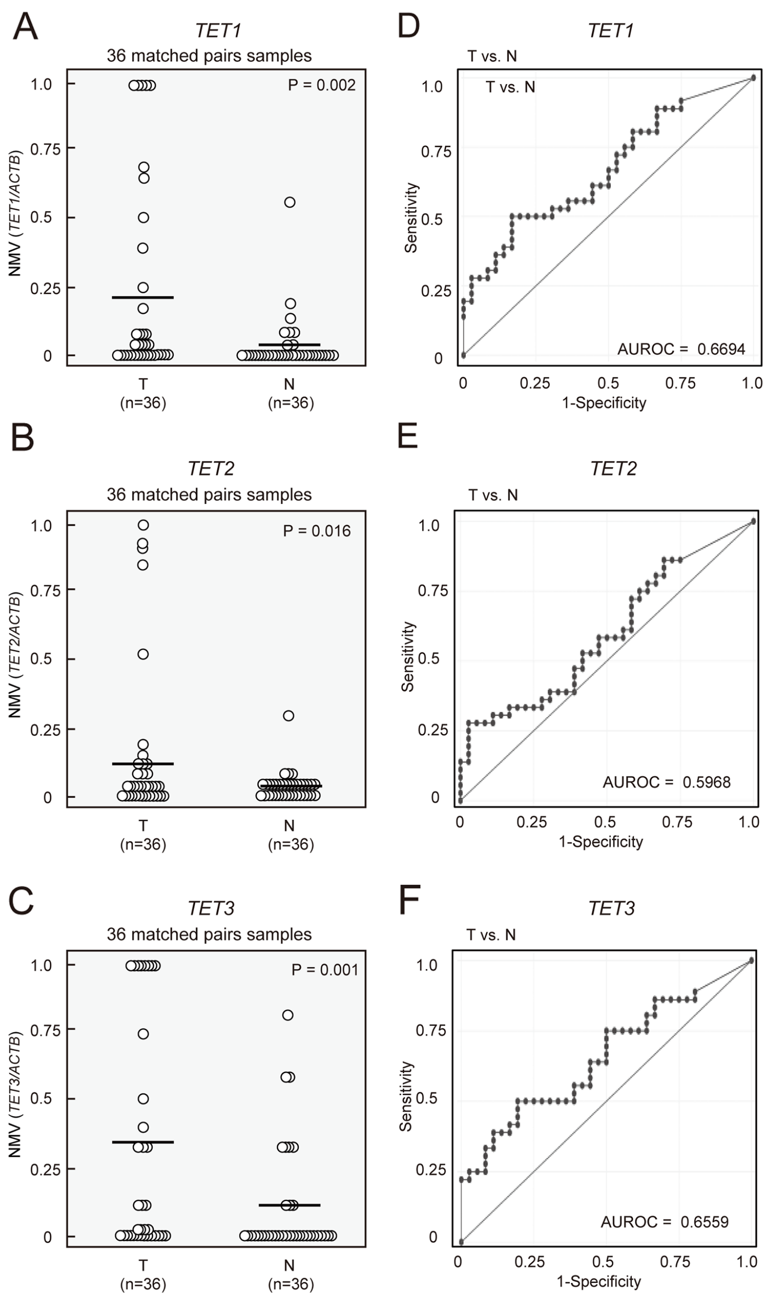

Supplementary Figure 1: Hypermethylation patterns in 36 matched pairs of head and neck tumors and adjacent normal mucosal tissues. *TET* methylation status in 36 matched pairs of head and neck tumor and normal tissue samples. The normalized methylation values (NMVs) for the **(A)** *TET1*, **(B)** *TET2*, and **(C)** *TET3* promoters were significantly higher in head and neck tumor tissues (T) than in paired adjacent normal mucosal tissue (N) (P = 0.002, 0.016 and 0.001, respectively). **(D)** The area under the ROC curve (AUROC) value for the *TET1* gene was 0.6694. At the cutoff value of 0.0471, the sensitivity was 50.0% and the specificity was 83.3%. **(E)** The AUROC value for the *TET2* gene was 0.5968. At the cutoff value of 0.1004, the sensitivity was 27.8% and the specificity was 97.2%. **(F)** Based on the ROC curve analysis, the AUROC value, sensitivity, specificity, and cutoff were 0.6559, 50.0%, 80.6%, and 0.1337, respectively, for *TET3*.

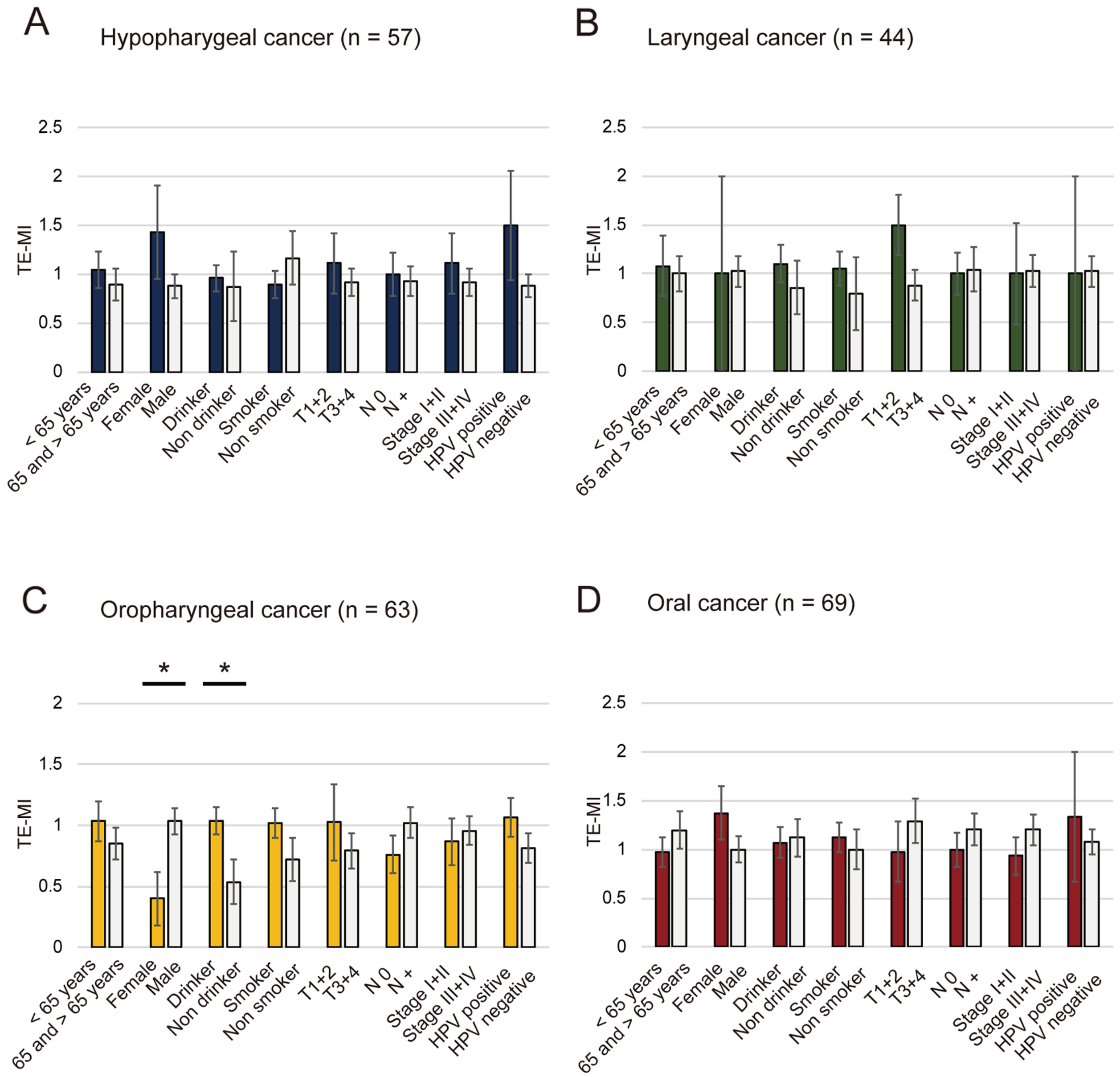

Supplementary Figure 2: Association between TE-MI and the selected clinical parameters. The mean TE-MI for the various groups was compared using Student's *t*-test. For associations between TE-MI and selected epidemiologic and clinical characteristics: **(A)** hypopharyngeal cancer, no differences were noted with regard to any of the clinical characteristics; **(B)** laryngeal cancer, no differences were noted with regard to any of the clinical characteristics; **(C)** oropharyngeal cancer, statistically significant differences were found for the associations between TE-MI and gender and alcohol consumption; **(D)** oral cavity cancer, no differences were noted with regard to any of the clinical characteristics. Mean and standard deviation are also indicated, and statistical comparisons between groups are depicted. $^{*}P < 0.05$ was considered to represent a statistically significant difference.

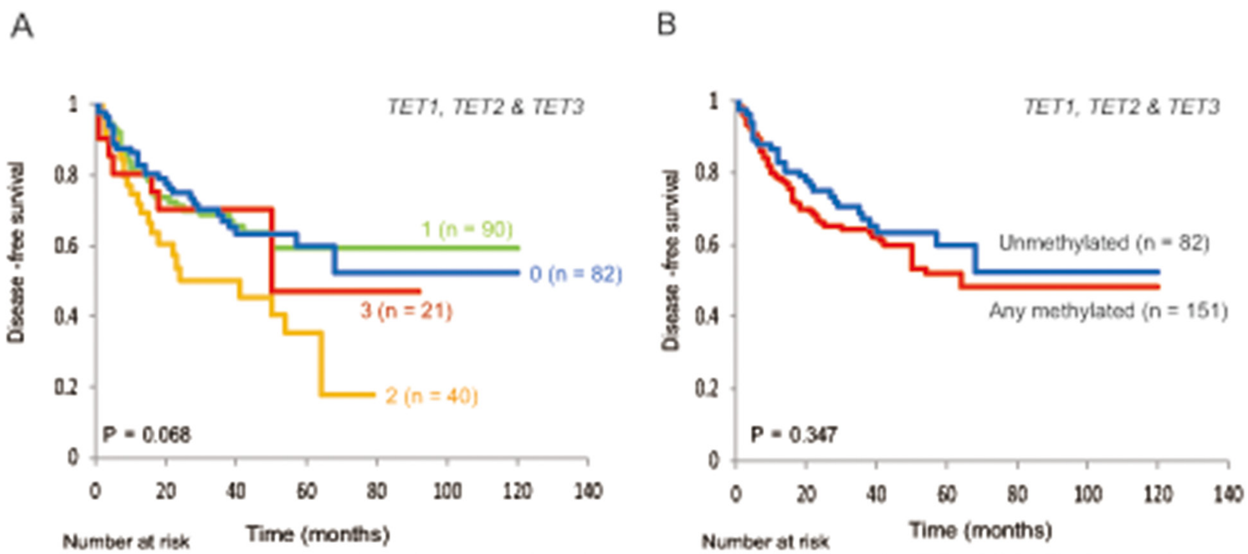

Number at risk

Time (Months)	0	20	40	60	80	100	120
0	82	61	34	16	3	1	1
1	90	61	36	27	6	2	2
2	40	19	11	6	0	0	0
3	21	14	8	3	2	0	0

Number at risk

Time (Months)	0	20	40	60	80	100	120
0	82	61	34	16	3	1	1
1-3	151	94	56	37	8	2	2

Supplementary Figure 3: Kaplan–Meier survival curves for patients with HNSCC according to *TET1*, *TET2* and *TET3* methylation status. **(A)** Joint analysis of the 3 genes (n = 233; P = 0.068). **(B)** Methylation of any the *TET* promoters versus no methylation of all three *TET* promoters (P = 0.347).

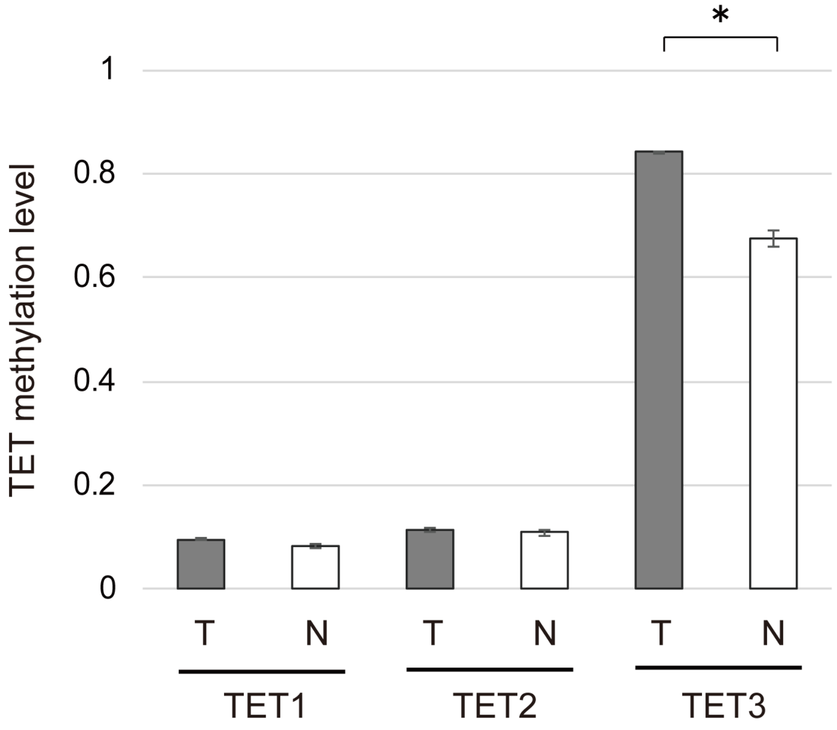

Supplementary Figure 4: DNA methylation data from the TCGA database. The DNA methylation data for *TET1*, *TET2* and *TET3* in HNSCC cancer were collected from the TCGA data portal (https://tcga-data.nci.nih.gov/tcga/) in November 2017.

Supplementary Table 1: *TET1, TET2* and *TET3* gene methylation status in tumor and normal mucosal tissues

Patient and tumor characteristics	Methylation status								
	TET1			*TET2*			*TET3*		
	methylation	unmethylation	*P*-value[a]	methylation	unmethylation	*P*-value[a]	methylation	unmethylation	*P*-value[a]
Tumor (233)	137	96	< 0.001	31	202	1	65	168	< 0.001
Normal (128)	48	80		19	109		10	118	

[a]Fisher's exact probability test. *P < 0.05.

Supplementary Table 2: Real time PCR primer list

QRT/QMSP	Gene	Forward/ Reverse	Sequence	Length (bp)
QRT	TET1	F	CCCTTGGAAATGCCATAGGAA	81
		R	GAGAGCCTGCTGGAACTGTTTG	
	TET2	F	GGCTGTTGGCCAGAGACTTA	117
		R	ATACCTGTAGGTGTTTGCCTGTTTA	
	TET3	F	GCCAACTTCAACATACCCTGGAC	81
		R	CACCTGGATGTGGGACTGTGTAA	
	GAPDH	F	GCACCGTCAAGGCTGAGAAC	138
		R	TGGTGAAGACGCCAGTCTCTA	
QMSP	TET1	F	ATCGGCGCGAGTTGGAAAGTT	103
		R	GACCCCAACTCACCGCTAACCG	
	TET2	F	CGCGGGTAACGGGATTTAAAG	123
		R	GTACCCTCGCTCTAACCCCCG	
	TET3	F	CGAGGGGGTGGAGATGGTCGAAAGAAAC	108
		R	CGTACGACGATTAATACAACT	
	ACTB	F	TGGTGATGGAGGAGGTTTAGTAAGT	133
		R	AACCAATAAAACCTACTCCTCCCTTAA	
